# HpDTC1, a Stress-Inducible Bifunctional Diterpene Cyclase Involved in Momilactone Biosynthesis, Functions in Chemical Defence in the Moss *Hypnum plumaeforme*

**DOI:** 10.1038/srep25316

**Published:** 2016-05-03

**Authors:** Kazunori Okada, Hiroshi Kawaide, Koji Miyamoto, Sho Miyazaki, Ryosuke Kainuma, Honoka Kimura, Kaoru Fujiwara, Masahiro Natsume, Hideaki Nojiri, Masatoshi Nakajima, Hisakazu Yamane, Yuki Hatano, Hiroshi Nozaki, Ken-ichiro Hayashi

**Affiliations:** 1 Biotechnology Research Center, The University of Tokyo, 1-1-1 Yayoi, Bunkyo-ku, Tokyo, 113-8657, Japan; 2 Graduate School of Agriculture, Tokyo University of Agriculture and Technology, 3-5-8 Saiwaicho, Fuchu, Tokyo 183-8509, Japan; 3 Department of Biosciences, Teikyo University, 1-1 Toyosatodai, Utsunomiya, 320-8551, Japan; 4 Department of Applied Biological Chemistry, The University of Tokyo, 1-1-1 Yayoi, Bunkyo-ku, Tokyo 113-8657, Japan; 5 Department of Biochemistry, Okayama University of Science, 1-1 Ridai-cho, Okayama 700-0005, Japan

## Abstract

Momilactones, which are diterpenoid phytoalexins with antimicrobial and allelopathic functions, have been found only in rice and the moss *Hypnum plumaeforme*. Although these two evolutionarily distinct plant species are thought to produce momilactones as a chemical defence, the momilactone biosynthetic pathway in *H. plumaeforme* has been unclear. Here, we identified a gene encoding *syn*-pimara-7,15-diene synthase (HpDTC1) responsible for the first step of momilactone biosynthesis in the moss. HpDTC1 is a bifunctional diterpene cyclase that catalyses a two-step cyclization reaction of geranylgeranyl diphosphate to *syn*-pimara-7,15-diene. *HpDTC1* transcription was up-regulated in response to abiotic and biotic stress treatments. *HpDTC1* promoter-GUS analysis in transgenic *Physcomitrella patens* showed similar transcriptional responses as *H. plumaeforme* to the stresses, suggesting that a common response system to stress exists in mosses. Jasmonic acid (JA), a potent signalling molecule for inducing plant defences, could not activate *HpDTC1* expression. In contrast, 12-oxo-phytodienoic acid, an oxylipin precursor of JA in vascular plants, enhanced *HpDTC1* expression and momilactone accumulation, implying that as-yet-unknown oxylipins could regulate momilactone biosynthesis in *H. plumaeforme*. These results demonstrate the existence of an evolutionarily conserved chemical defence system utilizing momilactones and suggest the molecular basis of the regulation for inductive production of momilactones in *H. plumaeforme*.

The moss *Hypnum plumaeforme* (Hypnaceae) is widely distributed in East Asia; it prefers sunlit locations and forms large homogeneous colonies on dry ground and rocks. *H. plumaeforme* has potent allelopathic activity, suppressing the growth of surrounding plants^1^. Momilactones, originally discovered in rice husks as allelochemicals and phytoalexins, are specialized metabolites that repress growth of neighbouring plants and defend against pathogens^2^,^3^. Momilactones accumulate in rice in response to both abiotic and biotic stresses, such as heavy metal treatment, ultra violet (UV) irradiation, and infection by the rice blast fungus^4^,^5^,^6^,^7^,^8^. Other than rice, the moss *H. plumaeforme* is the only plant species known to produce momilactones. We previously found that momilactones function as allelochemicals in this moss^1^. As a defence response to infection by pathogens such as *Botrytis cinerea*, the moss *Physcomitrella patens* exhibits fortification of the plant cell walls via incorporation of phenolic compounds^9^. Momilactones in *H. plumaeforme* accumulate in response to various stresses, including UV irradiation and heavy metal treatment^10^. These studies suggest that momilactone production could be a defence reaction in *H. plumaeforme*, in addition to providing allelopathic activity against other plants.

The biosynthetic pathway of momilactones and its corresponding genes have been extensively investigated in rice because of the similarity to the biosynthetic pathway of gibberellins (GAs), tetracyclic diterpene plant hormones (Fig. 1). In angiosperms, the cyclic diterpene skeleton, found in metabolites such as *ent*-kaurene, a precursor of GA, is derived from geranylgeranyl diphosphate (GGDP) by two different cyclases, *ent*-coparyl diphosphate synthase (*ent*-CPS) that belongs to type B cyclase and *ent*-kaurene synthase (KS) catalysing the type A cyclization of *ent*-copalyl diphosphate (*ent-*CDP). Rice also produces the cyclic diterpene skeletons of phytoalexins, momilactones, oryzalexins, and phytocassanes from GGDP by two different type B and A cyclases. In rice, *syn*-copalyl diphospate (*syn*-CDP) synthase (OsCPS4) and *syn*-pimara-7,15-diene synthase (OsKSL4) are involved in momilactone biosynthesis^11^. A key intermediate in the momilactone biosynthetic pathway, *syn*-pimara-7,15-diene, is further oxidized by cytochrome P450 enzymes and dehydrogenase to yield the active substances, that is, momilactones A and B^12^,^13^. The expression of all genes in momilactone biosynthesis is coordinately induced in response to both biotic and abiotic stresses, including blast fungus infection and heavy metal treatment^4^,^14^.

In contrast to vascular plants, the moss *P. patens* produces *ent*-kaurene via a bifunctional *ent*-kaurene synthase, PpCPS/KS, that is similar to fungal bifunctional *ent*-kaurene synthases^15^. PpCPS/KS has both type B and A activity in a single polypeptide and is the sole diterpene cyclase in the *P. patens* genome (Fig. 1)^15^. To date, other diterpene cyclase genes have not been identified from the mosses. Additionally, the molecular basis for the biosynthesis and physiological function of phytoalexins and allelochemicals in the moss has not been demonstrated. Therefore, the discovery of biosynthesis genes of momilactones in the moss would shed light on the ancient defence system of early land plants.

To dissect the biosynthetic pathway of momilactone and its regulatory mechanism in response to stress, we cloned the gene for *syn*-pimaradiene synthase (*HpDTC1*, *H. plumaeforme* diterpene cyclase 1), a key first step enzyme in momilactone biosynthesis. The *HpDTC1* gene encodes a bifunctional enzyme catalysing two sequential cyclization steps from GGDP to *syn*-pimara-7-15-diene via *syn*-CDP as the reaction intermediate. *HpDTC1* showed inductive expression under treatments with CuCl_2_, chitosan, and pathogen infection. Similar to *HpDTC1*transcriptional regulation in *H. plumaeforme,* the findings for transgenic *P. patens*, expressing an *HpDTC1* promoter-GUS construct (*HpDTC1::GUS*), demonstrated that the *HpDTC1* promoter is up-regulated by both abiotic and biotic stress treatments. An allelopathy activity assay showed that *H. plumaeforme* is highly resistant to extracts from *H. plumaeforme* and momilactone B at the active concentration for other types of mosses and liverworts. Additionally, the extract from *H. plumaeforme* showed phytoalexin activity against *B. cinerea* and protected the gametophore from infection by the pathogen. Our results aid in understanding the momilactone biosynthetic pathway in the moss and the molecular basis of an evolutionarily conserved chemical defence system between mosses and angiosperms utilizing momilactones.

## Results

### Identification of *H. plumaeforme* diterpene cyclase that is responsible for momilactone synthesis

**Figure 2:**
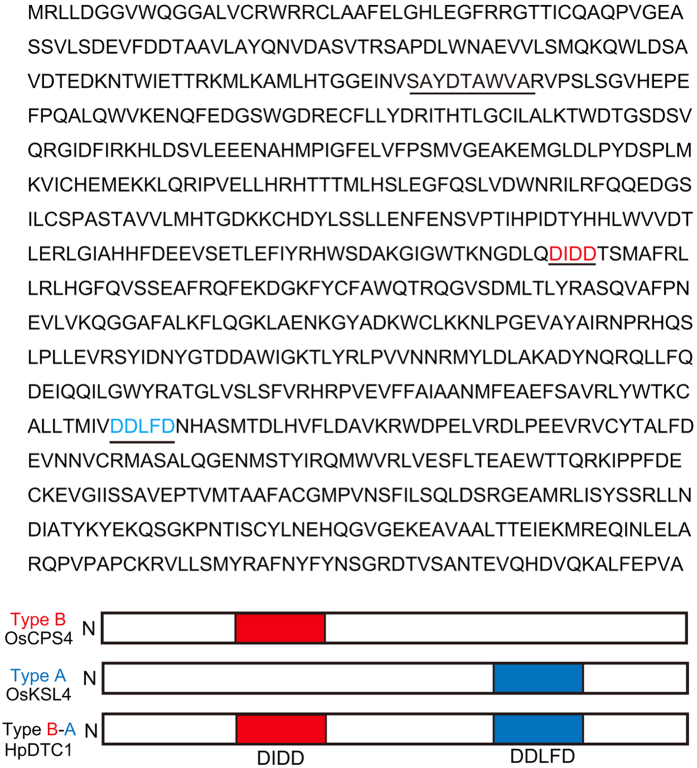
Amino acid sequence of HpDTC1 and active site motifs, DIDD and DDLFD. Three diterpene-specific motifs (SxYDTAWVA, DxDD, and DDxxD) are underlined. The DxDD motif in type B cyclase is responsible for the cyclization activity of GGDP to form CDP as a product. The DDxxD in type A cyclase is responsible for the cyclization of CDP to yield a cyclic diterpene hydrocarbon. Type B-A cyclase, HpDTC1, has two cyclization motifs in a single polypeptide and functions as a bifunctional cyclase with both type B and A cyclization activity.

Production of momilactones in *H. plumaeforme* has been reported to be highly induced by abiotic stresses, such as UV irradiation and heavy metals^10^. A key intermediate for momilactone biosynthesis in rice is *syn*-pimara-7,15-diene produced from GGDP. OsKSL4, a *syn*-pimara-7,15-diene synthase, is also induced by biotic and abiotic stresses in rice^12^. To identify the *syn-*pimaradiene synthase in *H. plumaeforme*, we screened for homologous genes of *P. patens CPS/KS* using next-generation sequencing of the cDNA library from sterile cultures of *H. plumaeforme* gametophores treated with CuCl_2_. We identified several CuCl_2_-upregulated contigs having homology to *PpCPS/KS* and gymnosperm bifunctional diterpene cyclases; one gene, designated *HpDTC1*, was the only diterpene cyclase gene in *H. plumaeforme* that was induced by CuCl_2_. Therefore, *HpDTC1* cDNA was amplified with RACE-PCR from a cDNA library from *H. plumaeforme* gametophores. The full-length *HpDTC1* cDNA was determined to be 2786 bp, encoding an 856 amino acid polypeptide (DDBJ ID: LC128408) as shown in Fig. 2. The N-terminal region (about 85 amino acids) was putatively assigned as a transit peptide region. The deduced HpDTC1 contained the three characteristic motifs for diterpene cyclases, SAYDTAWVA, DIDD, and DDLFD motifs (Fig. 2). Both active site motifs for type B (DxDD) and type A (DDxxD) cyclases indicated that HpDTC1 would function as a bifunctional diterpene cyclase similar to *PpCPS⁄KS*, gymnosperm diterpene synthase, and fungal CPS⁄ KS (see Supplementary Fig. S1). *HpDTC1* showed identity to *PpCPS⁄KS* (43%), *JsCPS/KS* (41%), *Selaginella moellendorffii* diterpene cyclases (DTCs) (38–41%), gymnosperm bifunctional DTCs (40–43%), plant CPSs (43–45%), and angiosperm KS and KS-like DTCs (30–32%). Phylogenetic analysis indicated that *HpDTC1* was most closely related to the gymnosperm bifunctional diterpene cyclase (see Supplementary Fig. S2).

### Functional characterization of HpDTC1 diterpene cyclase

**Figure 3:**
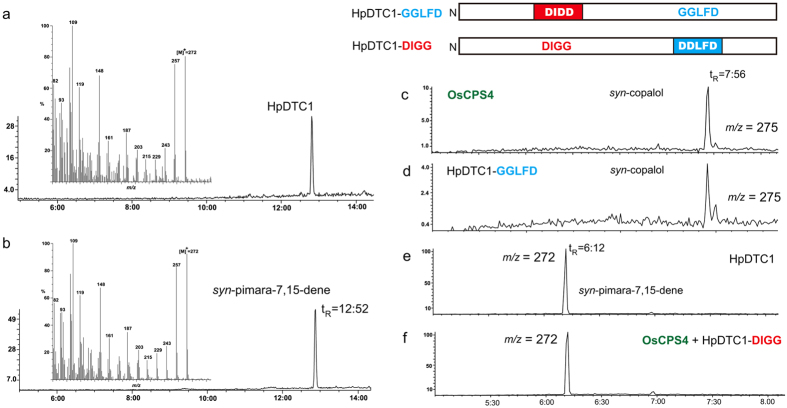
HpDTC1 is a bifunctional *syn*-pimaradiene synthase. GC-MS chromatograms of enzymatic products. (**a**) HpDTC1 product and (**b**) authentic sample of *syn*-pimara-7,15-diene. (**c**) *syn*-copalol derived from *syn*-CDP, product of OsCPS4. (**d**) The product of the HpDTC1-GGLFD mutant enzyme was identical to *syn-*copalol. (**e**) *syn*-pimara-7,15-diene, HpDTC1 product. (**f**) The product from the co-reaction of OsCPS4 and HpDTC1-DIGG enzyme was identical to *syn*-pimara-7,15-diene. The selected ion was monitored at *m⁄ z* 272 (**a**,**b**,**e**,**f**) or *m/z* 275 (**c,d**) to detect cyclized diterpenes. The reaction product, *syn*-CDP in (**c**,**d**) was detected as alcohol after dephosphorylation by calf intestinal alkaline phosphatase.

HpDTC1 showed two aspartate-rich motifs (DxDD and DDxxD) for active sites responsible for the sequential cyclization reaction of bifunctional diterpene cyclase^16^,^17^. *ent*-Kaurene synthase, *PpCPS/KS*, was the sole diterpene synthase gene identified from the mosses. PpCPS/KS is a bifunctional diterpene cyclase that catalyses the direct cyclization reaction of GGDP to yield *ent*-kaurene^15^. HpDTC1 is predicted to catalyse the cyclization of GGDP to give a tricyclic diterpene product via *syn*-CDP as the reaction intermediate. The recombinant HpDTC1 enzyme was expressed in *E. coli* as a His_6_-tag fusion protein and used for the enzyme assay using GGDP as a substrate. GC-MS analysis of the reaction products of HpDTC1 showed a single product peak on a GC-MS chromatogram, and the MS spectrum and retention time (Rt = 12.52 min) of the HpDTC1 product were identical to those of an authentic sample of *syn*-pimara-7,15-diene (Fig. 3a,b). This result demonstrated that HpDTC1 functions as bifunctional *syn*-pimaradiene synthase. In rice, diterpene phytoalexins, that is, momilactones, are biosynthesized from *syn*-CDP; in contrast, similar diterpene phytoalexins, that is, oryzalexins and phytocassanes, are synthesized from *ent-*CDP produced by OsCPS1/2^11^. To confirm the reaction intermediate of HpDTC1, we performed a mutational study on the HpDTC1 enzyme. The first aspartate-rich motif, DIDD, is required for the cyclization of GGDP to yield CDP (type B activity), whereas the second motif, DDLFD, is essential for the cyclization of CDP (type A activity)^18^. The loss-of-function mutation related to type A activity of HpDTC1 (DDLFD to GGLFD) resulted in the accumulation of an intermediate *syn*-CDP that was identical to *syn*-CDP produced by OsCPS4, a *syn*-CDP synthase (Fig. 3c,d; see Supplementary Fig. S3). The mutation in the first motif, DIDD (to DIGG), for type B activity disrupted the initial cyclase activity for GGDP in HpDTC1. Co-incubation with OsCPS4 and the type B mutant of HpDTC1 (DIGG) complemented the impaired type B activity of the DIGG mutant to produce *syn*-pimara-7,15-diene as the product, similar to the HpDTC1 wild type enzyme (Fig. 3e,f; see Supplementary Fig. S3). These findings clearly demonstrate that HpDTC1 is a bifunctional diterpene cyclase utilizing *syn*-CDP as a reaction intermediate.

### *HpDTC1* expression induced by both abiotic and biotic stresses correlates with the regulation of momilactone biosynthesis

In rice, momilactone biosynthesis is elevated in response to various biotic and abiotic stresses. Consistent with the induction of momilactone biosynthesis, the expression of *syn*-pimaradiene synthase (*OsKSL4*) was highly induced by these stresses^11^,^19^. *H. plumaeforme* also accumulated momilactones in response to abiotic stresses, such as UV irradiation and heavy metals. To examine the abiotic stress-induced expression of *HpDTC1,* we assessed *HpDTC1* transcriptional regulation and accumulation of momilactones in the gametophore under CuCl_2_ treatment. The gametophore culture was incubated in 500 μM CuCl_2_ and then the *HpDTC1* transcript level was determined at regular intervals by quantitative RT-PCR (qRT-PCR). *HpDTC1* transcript level was normalized to the transcript level of *HpACT3*, a housekeeping actin gene. Momilactones A and B in the gametophore were quantified by liquid chromatography with electrospray ionization tandem mass spectrometry (LC-MS/MS).

**Figure 4:**
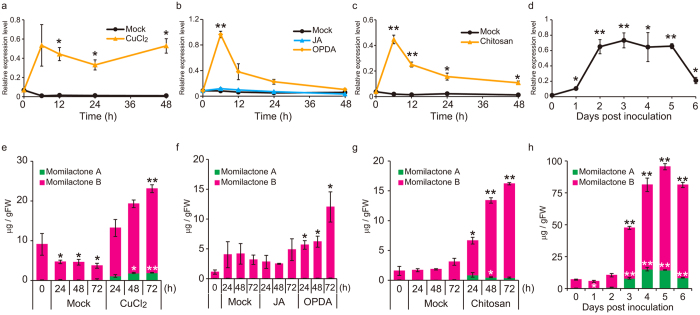
*HpDTC1* expression and momilactone production is induced by abiotic and biotic stresses. (**a**–**d**) qRT-PCR was performed using HpDTC1 specific primers on total RNA from *H. plumaeforme* gametophores treated with (**a**) CuCl_2_ (500 μM), (**b**) OPDA, 12-oxo phytodienoic acid, (100 μM) and jasmonic acid (100 μM), (**c**) chitosan (500 μg/mL) for the indicated times, and (**d**) the pathogenic fungus, *Botrytis cinerea*. The fungus *B. cinerea* was inoculated onto BCDATG agar plates of *H. plumaeforme* gametophores and then incubated for the indicated number of days. As an internal standard, the *H. plumaeforme* actin gene, *HpACT3*, was amplified by qRT-PCR and then the *HpDTC1* transcription level was normalized against the *HpACT3* level. (**e–h**) Momilactones A and B in *H. plumaeforme* gametophores treated with stresses were measured by LC-MS/MS. Data are presented as mean ± standard error. (**a**–**c**,**e**–**h**) n = 3, (**d**) n = 4, *p < 0.05, **p < 0.01.

As shown in Fig. 4a, abiotic stress by CuCl_2_ treatment dramatically induced *HpDTC1* expression within 6 h and the expression remained at a high level for 48 h after the treatment. Consistent with this *HpDTC1* induction, momilactones A and B also accumulated to a high level with CuCl_2_ treatment (Fig. 4e). Similar to the induction of momilactone synthesis in rice, *H. plumaeforme* was reported to induce momilactone production in response to jasmonic acid (JA), a signalling molecule involved in the defence response of vascular plants^10^. However, under our experimental conditions for the cultured gametophores, enhancement of accumulation of momilactones was barely detected with JA treatment in the sterile culture of *H. plumaeforme* gametophores (Fig. 4f). Consistent with the lack of response of momilactone production to JA, *HpDTC1* expression showed almost negligible change with JA treatment (Fig. 4b). 12-Oxo-phytodienoic acid (OPDA), a biosynthetic precursor of JA, functions in activation of the defence response in the moss^9^,^20^. Thus, we also examined the effect of OPDA on the expression of *HpDTC1* and production of momilactones. As shown in Fig. 4b, we found that OPDA clearly induced *HpDTC1* gene expression at 6 h after the treatment, and increased accumulation of momilactones was also detected 72 h after the treatment. Taken together, the response of *HpDTC1* expression and momilactone production to CuCl_2_ and OPDA treatment suggests that *HpDTC1* expression, triggered by these abiotic stresses, correlates well with momilactone accumulation.

We next examined the effects of biotic stress on *HpDTC1* expression. Biotic elicitors activate the defence system of rice and induce the expression of diterpene cyclase genes for phytoalexins, leading to the accumulation of diterpene phytoalexins, including momilactones^21^,^22^. Chitosan is also reported to induce defence-related responses in the moss^23^. Therefore, *H. plumaeforme* gametophores were incubated with chitosan. In addition, *B. cinerea*, a necrotrophic plant pathogenic fungus, infects the gametophores of moss plants and causes disease symptoms^9^. *B. cinerea* was inoculated onto *H. plumaeforme* gametophores and then incubated for 6 days (see Supplementary Fig. S4). *HpDTC1* expression levels in the chitosan-treated or *B. cinerea*-infected gametophores were analysed by qRT-PCR. Both chitosan treatment and *B. cinerea* infection up-regulated *HpDTC1* expression (Fig. 4c,d) and accumulation of momilactones was significantly induced by these biotic stresses (Fig. 4g,h). These results suggest that the *HpDTC1* gene is stress-responsive not only to abiotic stress, but also to biotic stress, and the expression of *HpDTC1* is part of the defence response through the production of phytoalexin momilactones in *H. plumaeforme.*

### *HpDTC1* expression is regulated by a common stress signalling pathway conserved in moss plants

**Figure 5:**
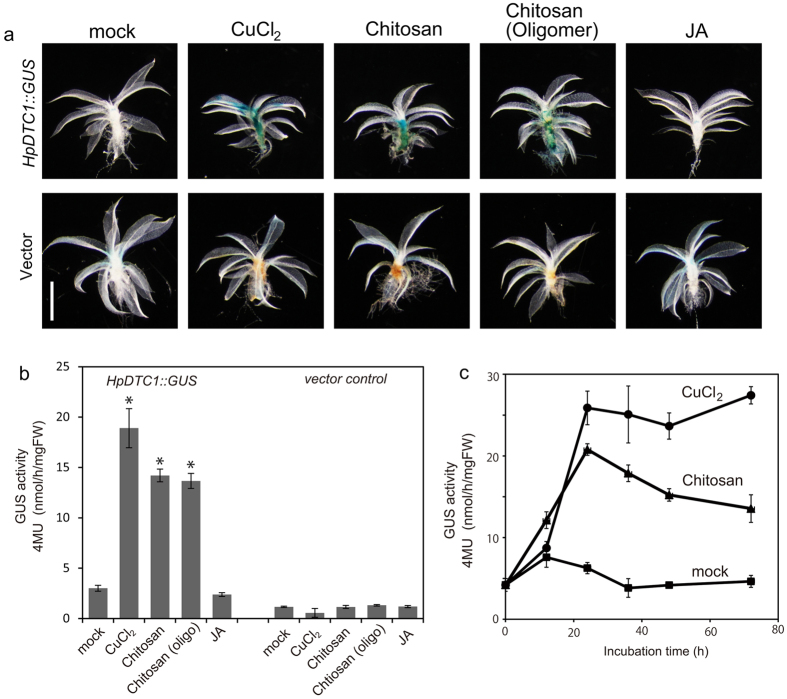
The *HpDTC1* promoter is regulated by abiotic and biotic stress responses in the moss *Physcomitrella patens.* (**a**) *HpDTC1 promoter::GUS* (*HpDTC1::GUS*) expression was histochemically visualized after 24 h stress treatment. The *HpDTC1::GUS* reporter was highly induced by CuCl_2_ (500 μM), chitosan (250 μg/ml), and chitosan oligomers (Average Mw = 4000–6000, 250 μg/ml), but not by jasmonate, JA, (100 μM) on leafy gametophores (upper panel). The vector control line did not respond to the stress treatments (lower panel). Scale bars: 1 mm. The quantitative data for *HpDTC1::GUS* reporter induction at 24 h treatment (**b**) by CuCl_2_ (500 μM), chitosan (250 μg/ml), chitosan oligomers (250 μg/ml), and JA (100 μM), and at the indicated time (**c**) by CuCl_2_ (500 μM) and chitosan (250 μg/ml). The GUS activity of *HpDTC1::GUS* gametophores was fluorometrically determined. Data are presented as mean ± standard deviation of biological replicates (*n* = 5 for (**b**), n = 4 for (**c**); *p < 0.001).

To assess whether the stress signalling pathway regulating *HpDTC1* expression is commonly conserved in other mosses, a GUS reporter assay for the *HpDTC1* promoter was performed in the model moss *P. patens*. The promoter region of *HpDTC1* was amplified by TAIL-PCR from genomic DNA and 3.4 kbp of the *HpDTC1* promoter was inserted into the GUS reporter plasmid, *pPIG1bNGGII*. The *HpDTC1promoter::GUS* construct (*HpDTC1::GUS*) was used to transform *P. patens* protoplasts by a conventional PEG-mediated method. The transgenic *HpDTC1::GUS* line showed very faint GUS basal expression in gametophores, similar to the control line (transformed with a promoter-less *pPIG1b-NGGII* vector) (Fig. 5a). In contrast, the protonema cells showed significant basal GUS expression compared to the control line (see Supplementary Fig. S5). The treatment of protonema cells with CuCl_2_ and chitosan did not strongly induce *HpDTC1::GUS* reporter expression (see Supplementary Fig. S5). However, consistent with *HpDTC1* expression in *H. plumaeforme*, the treatment of gametophores with CuCl_2_ dramatically induced *HpDTC1::GUS* expression in *P. patens* (Fig. 5a,b). Chitosan and its oligomers, functioning as elicitors, and the chitosan-inducible peroxidase, Prx34, play a crucial role in the basal defence system against fungal invasion in *P. patens*^23^. Consistent with the elicitation of the *P. patens* defence response with chitosan, the treatment with chitosan and its oligomer greatly upregulated GUS reporter expression in gametophores (Fig. 5a,b). *HpDTC1::GUS* induction reached its maximum at 24 h after exposure to these stress conditions (Fig. 5c). However, jasmonic acid had little effect on *HpDTC1::GUS* expression in *P. patens*, similar to *H. plumaeforme* (Fig. 5a,b). In contrast, OPDA treatment weakly but clearly induced *HpDTC1* promoter activity, as seen in the expression of *HpDTC1* in OPDA-treated *H. plumaeforme* (see Supplementary Fig. S6), suggesting that OPDA plays a role in common defence signalling in the mosses.

**Figure 6:**
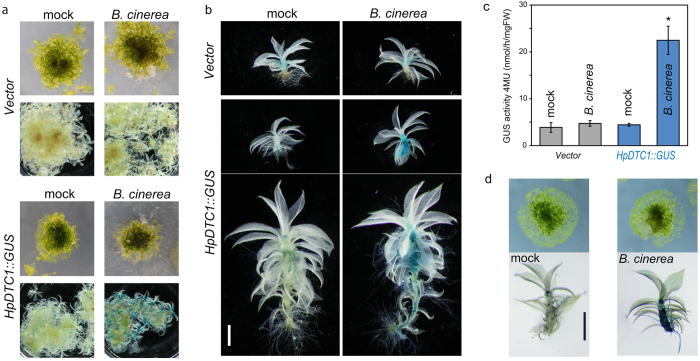
The *HpDTC1* promoter is induced by *Botrytis cinerea* infection in the moss *Physcomitrella patens.* (**a,b**) The pathogenic fungus, *B. cinerea*, was inoculated onto colonies of *HpDTC1::GUS* and vector control lines and then the culture plate was incubated for 4 days at 24 °C. The *HpDTC1::GUS* plants were histochemically stained. Scale bars: 1 mm. (**c**) The quantitative data for *HpDTC1::GUS* reporter induction by *B. cinerea* infection after 4 days incubation. The GUS activity was fluorometrically determined. Data are presented as mean ± standard deviation of biological replicates. (*n* = 5–8, *p < 0.01.). (**d**) Application of sterilized mycelia of *B. cinerea* induced *HpDTC1::GUS* reporter expression. The mycelia were placed on a *HpDTC1::GUS* colony and then incubated for 4 days at 24 °C.

The response of *HpDTC1* promoter expression under biotic stress was also examined with fungal infection; *B. cinerea*^24^ or *Pythium irregulare*^25^ was inoculated onto a *H. plumaeforme* colony and then cultured for 4 days. Consistent with the induction of *HpDTC1* in *H. plumaeforme*, *HpDTC1::GUS* expression was highly induced by fungal infection of both *B. cinerea* (Fig. 6a–c) and *P. irregulare* (see Supplementary Fig. S7), while GUS activity was not observed in the vector control line. Additionally, sterilized mycelia of *B. cinerea* also activated *HpDTC1::GUS* expression, suggesting an elicitor component from the mycelia would activate the defence response in *P. patens* (Fig. 6d). This evidence suggests that a common defence signalling pathway, conserved among mosses, would function in defence-related gene expression, including *HpDTC1* in *H. plumaeforme.*

### Physiological role of momilactones in defence response of *H. plumaeforme*

**Figure 7:**
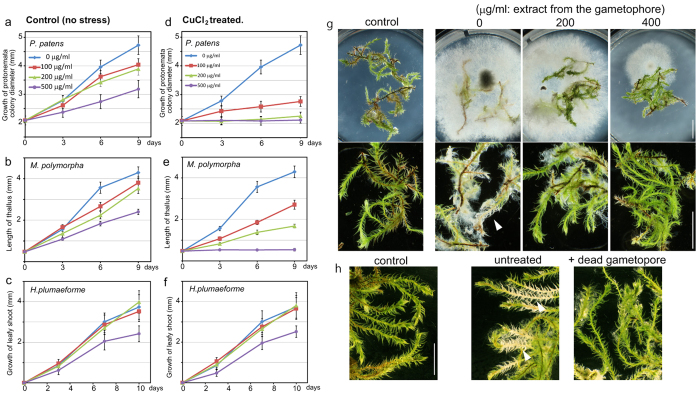
Physiological function of momilactone produced by *H. plumaeforme*. (**a–f**) The allelopathic effects of inductively produced momilactones on the moss *P. patens* (**a,d**), liverwort *Marchantia polymorpha* (**b,e**), and *H. plumaeforme* (**c,f**). The acetone extract was prepared from *H. plumaeforme* gametophores treated with or without CuCl_2_. The plants were cultured on BCDATG agar plates containing the extract at the indicated concentration and growth recorded at the indicated times. Data are presented as mean ± standard deviation of biological replicates (*n* = 15 for (**a,d**), *n* > 18 for (**b,e**), *n* = 8 for **c,f**). (**g**) Effects of *H. plumaeforme* extract on the growth of the pathogenic fungus, *B. cinerea*. The mycelia were inoculated onto the BCDATG plate containing *H. plumaeforme* acetone extract and the gametophores. The inoculated plate was incubated for 4 days. (**h**) Effects of *H. plumaeforme* gametophores on the growth of *B. cinerea*. *H. plumaeforme* gametophores were dried at 70 °C and then agar plates were prepared with the dead gametophores suspended in BCDATG agar (10% w/v). Fresh *H. plumaeforme* gametophores were placed on the agar plates; then, the mycelia were inoculated and incubated for 7 days. Arrowheads indicate the cells damaged by the pathogenic infection.

Momilactones are exuded as allelopathic substances from the roots of rice^3^. The moss *H. plumaeforme* forms large homogeneous colonies in dry areas. Momilactones could be accumulated as allelopathic substances in the gametophores to prevent the invasion of the moss colony by competing plant species. To confirm the allelopathic role of momilactones in *H. plumaeforme,* the plant growth inhibitory activity of the extract from the gametophore was measured (Fig. 7a–f; see Supplementary Fig. S8). The extract from stress-induced gametophores (Fig. 7d,e) showed more potent growth inhibition on *P. patens* and *M. polymorpha* than the control extract (Fig. 7a,b). In contrast, *H. plumaeforme* was highly resistant to both extracts at 200 μg/ml (Fig. 7c,f). Additionally, pure momilactone B showed no effects on the growth of *H. plumaeforme* at 100 μM, but showed potent inhibition on the growth of *M. polymorpha* at 5 μM (see Supplementary Fig. S9). This evidence supports the allelopathic role of momilactones in the formation of large homogeneous colonies of *H. plumaeforme.*

*HpDTC1* expression in the moss was induced by the chitosan elicitor and *B. cinerea* infection, suggesting the production of momilactones is a defence response to pathogenic attack. To investigate the role of momilactone as a phytoalexin in *H. plumaeforme,* we examined the protective activity against *B. cinerea* infection of momilactone produced by *H. plumaeforme*. The acetone extract from the stress-induced gametophytes showed potent inhibitory activity on the growth of *B. cinerea*, implying momilactone plays a role as a phytoalexin of *H. plumaeforme* to protect from the pathogenic attack of *B. cinerea* (Fig. 7g). In the natural world, *H. plumaeforme* forms large and dense colonies composed of stacked gametophytes^1^. Momilactones were found in the soil covered by *H. plumaeforme* colonies, suggesting *H. plumaeforme* accumulates momilactones in response to stresses and releases them as phytoalexins to protect from pathogenic attack from the soil. The momilactones released from wounds or dead cells in the stacked gametophyte colony could play a role in the defence system of *H. plumaeforme*. In our experiment, living gametophytes were inoculated with *B. cinerea* on a medium containing stress-treated dead gametophytes (Fig. 7h; see Supplementary Fig. S10). The application of stress-treated dead gametophytes to the medium repressed the growth of *B. cinerea* and reduced the damage of *H. plumaeforme* by the infection (Fig. 7h), implying that the stacked gametophyte colony could play a role in the chemical defence system of moss against the pathogenic fungus. These results support the physiological function of momilactones as both phytoalexins and allelopathic substances in the defence response of *H. plumaeforme.*

## Discussion

In this study, we identified a stress-inducible *H. plumaeforme* diterpene cyclase gene, *HpDTC1*, encoding *syn*-pimaradiene synthase, which catalyses key steps of momilactone biosynthesis to yield *syn*-pimara-7,15-diene from GGDP via *syn*-CDP as the reaction intermediate. HpDTC1 is a bifunctional enzyme consisting of type B cyclase that is responsible for *syn*-CDP synthesis and type A activity for *syn*-pimaradiene synthesis. In angiosperms, cyclic diterpenes such as *ent-*kaurene are key intermediates of gibberellin biosynthetic pathways and *syn*-pimaradiene is a precursor for momilactone. These precursors are synthesized by the successive reactions of monofunctional type B and type A cyclases^11^,^19^,^26^. In gymnosperms and lycophytes, *ent-*kaurene is synthesized by two monofunctional diterpene cyclases, *ent-*CPS (type B) and KS (type A)^27^, whereas other cyclic diterpenes for secondary metabolites, such as miltiradiene, pimaradiene, levopimaradiene, and abietadiene, are produced by bifunctional type B-A diterpene cyclases^28^,^29^. In contrast, the *ent*-kaurene synthase, PpCPS/KS, in the moss has been identified as a bifunctional type B-A cyclase that is involved in the biosynthesis of diterpene-type growth regulators for protonema development in *P. patens*^14^. PpCPS/KS is the sole diterpene synthase in the moss *P. patens* and other DTCs have not been identified from other mosses. HpDTC1, therefore, is the first diterpene synthase identified that is responsible for the biosynthesis of specialized metabolites in the mosses. In the defence response of gymnosperms, bifunctional abietadiene synthase was induced by wound stress to produce diterpene resin acids as protective substances^17^. Consistent with the role of the bifunctional DTC in the gymnosperm defence system, phylogenetic analysis showed HpDTC1 is more closely related to the gymnosperm bifunctional DTCs than to moss (PpCPS/KS) and liverwort (JsCPS/KS) *ent*-kaurene synthases, implying HpDTC1 could be the prototype of cyclases for specialized diterpenes for defence responses in the land plants (see Supplementary Fig. S2).

Momilactone production was reported to be induced by various stresses, including UV irradiation and some chemical elicitors, such as JA, CuCl_2_, and phosphatase inhibitors^10^. We confirmed significant induction of momilactone production and *HpDTC1* expression in our study in response to abiotic stress treatments, except for the JA treatment. The lack of responsiveness of momilactone synthesis to JA contradicts a previous study by Kato-Noguchi *et al.*^10^. However, it has been reported that endogenous JA is not consistently detected at physiologically active levels in mosses, including *P. patens* and *M. polymorpha*, and no obvious physiological effects on plant development could be observed with JA treatment^30^,^31^. In fact, JA accumulation in wound/CuCl_2_-treated *H. plumaeforme* was undetectable by LC-MS/MS (see Supplementary Fig. S11). In contrast, we found that OPDA, a precursor of JA, has the ability to activate *HpDTC1* expression and momilactone production in *H. plumaeforme.* Notably, OPDA has been reported to act as a signalling molecule to induce defence responses in *P. patens*^9^. These observations suggest that the active signalling molecules in moss might be OPDA or its metabolites, and differ from those in vascular plants. Further evidence is needed to demonstrate how OPDA or its metabolites can activate momilactone production in *H. plumaeforme*.

Currently, the molecular basis of defence responses in the moss is not well understood. However, there have been some reports of defence responses in mosses. For example, Lehtonen *et al.* reported that type III peroxidase (Prx34), which is known as a PR protein involved in plant defence, is rapidly induced in response to chitosan treatment. Further, the knock-out of the *Prx34* gene in *P. patens* confers the hyper-sensitive phenotype to pathogenic fungus infection^23^. In addition, the PR genes in *P. patens*, that is, those encoding phenylalanine ammonia-lyase, chalcone synthase and lipoxygenase, were up-regulated by the pathogenic attack of *B. cinerea* and *P. irregulare*^30^. Our results also showed that biotic stresses, such as chitosan treatment and *B. cinerea* infection, clearly induced both momilactone accumulation and *HpDTC1* expression, suggesting that momilactone biosynthesis in *H. plumaeforme* is part of the defence response to pathogen attack. Our findings here in *H. plumaeforme*, together with previous reports in *P. patens*, strongly support that defence responses leading to the expression of *PR* genes, *HpDTC1* and *Prx34*, are a common strategy in mosses to resist attacks by pathogens. *H. plumaeforme* could have evolved the ability to use momilactone synthesis as a chemical weapon against pathogens. In addition, momilactones function as allelochemicals, preventing growth of neighbouring plants. Thus, *H. plumaeforme* has a great growth advantage when facing biotic environmental stresses like pathogens and competition with other plants.

In our histochemical GUS staining analysis, the transgenic *HpDTC1::GUS* line showed GUS reporter gene induction when the gametophore was treated with CuCl_2_, chitosan, and infection by *B. cinerea*. We also found that the expression of *HpDTC1* has different responses in different developmental stages. Protonema cells are an early developmental stage of moss that forms a highly branched filamentous structure. When we tested GUS expression in protonema cells of *HpDTC1::GUS* reporter lines with treatments of CuCl_2_ and chitosan, in contrast to the gametophore, high basal expression and no obvious induction of the GUS reporter gene were observed in whole protonema cells (see Supplementary Fig. S12). This result is consistent with *HpDTC1* expression and momilactone production in *H. plumaeforme* protonema cells, showing uniform momilactone production and *HpDTC1* expression, even in the treatment with the chitosan elicitor (see Supplementary Fig. S5). This finding also suggests that defence-related transcriptional regulation would only be active in the gametophore. Moreover, our results indicate that the *HpDTC1* promoter is active in the model moss plant, *P. patens.* This result indicates that regulatory mechanisms of *HpDTC1* expression could be conserved among mosses, i.e., similar transcriptional regulation might function in *HpDTC1* expression in both mosses.

In *P. patens*, oxylipins could function in the defence response and regulate plant development, but the signalling component(s), specifically transcriptional regulators in oxylipin-regulated pathways, are still not understood. For momilactone synthesis and its transcriptional regulation of biosynthetic gene expression in rice, several transcription factors regulating momilactone synthesis have been identified. A bZIP-type transcription factor, OsTGAP1^32^,^33^, a bHLH-type transcription factor, DPF^14^, and WRKY-type transcription factors, OsWRKY53 and OsWRKY45^34^,^35^, have been shown to function as positive regulators of momilactone biosynthetic gene expression, whereas OsbZIP79 and OsWRKY76 have potential activity as negative regulators^36^,^37^. In *H. plumaeforme*, many types of transcription factors (ERF, bHLH, and WRKY-type) were induced by CuCl_2_ treatment in our RNA-seq result (see Supplementary Fig. S13). However, these transcription factors did not have high similarity to rice orthologs of the regulatory factors for momilactone synthesis. It seems that different factors are involved in defence signalling leading to momilactone production in rice and *H. plumaeforme* (both momilactone producers), although mosses would likely possess a common system for transcriptional regulation of defence-related gene expression. Further investigation of the transactivation activity of these factors to the *HpDTC1* promoter will provide possible candidates for modulating stress-responsive expression of the momilactone biosynthetic *HpDTC1* gene.

Momilactones, which are inductively produced in *H. plumaeforme* and rice, are typical specialized metabolites for chemical defence strategies in these plants. When and how these two evolutionarily distinct species acquired the ability to produce the identical specialized diterpenes and to regulate their biosynthesis comprise one of the great mysteries of science. Further, how rice and *H. plumaeforme* resist allelopathic substances, such as momilactones, when the growth of many other plants is inhibited by momilactones, is an interesting question. The mode of action and the candidates for factors enabling resistance to momilactones are not known at present. Further exploration of candidate genes responsible for such resistance mechanisms will provide insight into allelopathic activity of and resistance mechanisms to momilactones. On-going whole genome analysis of *H. plumaeforme* might reveal a broad picture of the evolutionary steps towards the momilactone biosynthetic pathway in plants.

## Methods

### Plant Materials and Growth Conditions

The wild-type strain of *Hypnum plumaeforme* was collected at Okayama, Japan, and sterilized with benzethonium chloride (0.1 w/v% aqueous solution). The sterilized gametophores were cultured on BCDATG agar medium^38^ under continuous white light at 24 °C. The wild-type strain of *Physcomitrella patens* subsp *patens* was cultured on BCDAT agar medium under continuous white light at 24 °C. *Marchantia polymorpha* was cultured on half-strength Gamborg’s B5 medium containing 8 g/L agar under continuous white light at 24 °C. For the stress treatment, media containing 8 g/L agar under continuous white light at 24 °C was used. For the stress treatment, the *H. plumaeforme* culture was incubated with BCDATG liquid media containing the chemicals and elicitors (jasmonic acid (Tokyo Kasei, Japan), low molecular weight chitosan (Aldrich, Japan), and chitosan oligosaccharide lactate (Aldrich, Japan)) or aqueous solution of copper (II) chloride dihydrate (Nacalai Tesque, Japan). Chitosan was dissolved in water by the addition of acetic acid, and final pH of the solution was adjusted to pH 6.0. The acetone extract of *H. plumaeforme* was prepared from the culture of gametophores incubated with or without 500 μM CuCl_2_ for 96 h. The gametophore was soaked in acetone for 24 h at 20 °C and then the organic layer was evaporated *in vacuo*. For the growth inhibitory assay, the plants were inoculated on the corresponding agar medium containing the acetone extract in a 6-well plate and cultured under continuous white light at 24 °C. Photographs of the plant were taken at regular intervals and the growth of the plant was analysed using ImageJ software. For the stress treatment of the *HpDTC1::GUS* reporter line, *P. patens* gametophores grown on BCDAT agar media for 3 weeks were incubated in BCDATG liquid media containing the chemicals and elicitors for 24 h under continuous white light at 24 °C. For the infection experiment, the necrotrophic plant fungus, *B. cinerea* MAFF237501 and *Pythium irregulare* MAFF237249, were obtained from the GeneBank of the National Institute of Agrobiological Sciences (Japan) and grown on potato dextrose agar medium. Agar blocks (1 mm) containing the mycelia were placed on a *P. patens* colony or *H. plumaeforme* gametophore on BCDATG agar plates. The plates were incubated under continuous white light at 24 °C.

### RNA-Seq

*H. plumaeforme* gametophores were treated with or without 500 μM CuCl_2_ for 8 h. Total RNA was extracted from the gametophore (500 mg) using the RNeasy Plant Mini Kit (QIAGEN, USA) according to the manufacturer’s instructions. The concentrations and qualities of RNA were determined with the Agilent Bioanalyser 2100 (Agilent Technologies, Inc., Santa Clara, CA, USA). RNA concentration was adjusted to 1 μg/μL, and ratios of 28S/18S were estimated to be higher than 2.10. Total RNA (9 μg per sample) was used for the following library generation and sequencing conducted by BGI Japan.

### Identification of HpDTC1 gene

The BLAST search of diterpene synthase against RNA-seq data revealed several contigs of diterpene cyclase homologous to the plant bifunctional terpene synthases PpCPS/KS (AB302933.1), JsCPS/KS (BAJ39816), and AgAs (AAK83563). Primers used for PCR and plasmid construction are shown in Supplementary Table S1. Based on the sequence information, *HpDTC1* cDNA fragments were amplified by PCR using internal primers, HpDTC1-FR1, and then both 5′ and 3′ ends of *HpDTC1* cDNA were amplified by RACE-PCR (Gene Racer, Invitrogen, Japan). The full length of *HpDTC1* cDNA was amplified by PCR (Phusion High-Fidelity DNA Polymerase) using HpDTC1_cDNA primers. The genomic sequence of HpDTC1 was confirmed by PCR and the *HpDTC1* promoter region was determined by four rounds of TAIL-PCR with arbitrary degenerate primers and gene specific primers. The 4.5 kbp HpDTC1 promoter region was amplified by PCR with PR-InF-HpDTC1 primers and subcloned into the *KpnI* and *HindIII* sites of pUC18 using the In-Fusion Cloning Kit (Clontech, Japan).

### Recombinant protein expression and functional analysis

The open reading frame lacking the region encoding the plastid transit peptide of *HpDTC*1 was ligated into the *KpnI* and *EcoRI* sites of the pCold I vector (Takara Bio, Japan) using two sets of primers, HpDTC1-ORF. The recombinant HpDTC1 protein was produced in *Escherichia coli* strain M-15 as a His_6_-tagged protein according to the manufacture’s protocol and our previous work^27^. Enzyme products were identified using a GC-MS system (Bu-25, Jeol, Tokyo, Japan) equipped with a DB-WAX capillary column (0.25 mm *ϕ*, length 30 m, and film thickness 0.25 μm; J&W Scientific).

Enzyme reactions and preparation of samples for analysis were performed as reported previously^27^. To identify an intermediate during the bifunctional cyclization reaction from GGDP to *syn*-pimara-7,15-diene, an amino acid-substituted mutant of HpDTC1 was prepared by PCR using two sets of primers (double-mutation-A and double-mutation-B in Supplementary Table S1) as described previously^27^,^39^. The products obtained from wild type HpDTC1 and the HpDTC1 mutant were determined by using a GC-MS system equipped with a DB-1 capillary column (0.25 mm *ϕ*, length 15 m, and film thickness 0.25 μm; J&W Scientific).

### RNA extraction and quantitative RT-PCR

Total RNA was extracted from *H. plumaeforme* gametophores treated with various elicitors at regular time intervals using a Sepasol-RNA I Super G (Nacalai Tesque, Japan) and subjected to cDNA synthesis using a PrimeScript RT reagent Kit with gDNA Eraser (Takara Bio, Japan). Quantitative RT-PCR (qRT-PCR) was performed using a Power SYBR Green PCR Master Mix (Applied Biosystems, CA, USA) for the *HpDTC1* and *HpACT3* genes on an ABI 7500 Fast Real-Time PCR System (Applied Biosystems) with standard mode. To calculate the transcript levels of characterized genes, the copy number of their mRNAs was determined by generating standard curves using a series of known concentrations of the target sequence. *HpACT3* (DDBJ ID: LC129863, homologous to *P. patens Actin3* gene (AAQ88110.1) found in RNA-seq) was used as an internal control to normalize the amount of mRNA. For each sample, the mean value from triplicate amplifications was used to calculate the transcript abundance. Sequences of PCR primers used for qRT-PCR analysis are provided in Supplementary Table S1.

### Measurements of momilactone accumulation

Moss samples were soaked in 2 ml extraction solvent (MeOH/H_2_O, 80:20 [v/v]) and incubated at 4 °C overnight. Then, 5 μL of the extract was subjected to phytoalexin measurement by LC-MS/MS. An Agilent 1200 separation module (Agilent Technologies, Palo Alto, CA, USA) equipped with a CAPCELL CORE C_18_ column (50 mm long, 2.1 mm in diameter; Shiseido, Tokyo, Japan) was used for HPLC analysis. The mobile phase consisted of 0.05% AcOH in H_2_O (solvent A) and 0.05% AcOH in MeCN (solvent B). Elution was conducted using a linear gradient from 40% to 60% solvent B over 10 min at a flow rate of 0.2 mL/min, and the eluate was monitored by an Agilent 6460 Triple Quadrupole mass spectrometer (Agilent Technologies, Palo Alto, CA, USA) using the positive ion mode. Electrospray conditions were as follows: capillary voltage, 3500 V; drying gas flow, 5 L/min nitrogen; drying gas temperature, 300 °C; nebulizer pressure, 45 psi; sheath gas temperature, 350 °C; and sheath gas flow, 11 L/min. Dwell time and fragmentor voltage were 200 ms and 135 V, respectively. The multiple reaction monitoring mode (MRM) was used in the MS/MS. MRM transitions were as follows: *m*/*z* 315.2/271.1 for momilactone A; *m*/*z* 331.2/269.1 for momilactone B.

### Plasmid construction and moss transformation

A 3.4 kbp fragment of the HpDTC1 promoter (DDBJ ID: LC128407) was amplified by PrimeSTAR Max DNA polymerase (Takara Bio, Japan) using the primer pair, PR-DTC1-GUS (Supplementary Table S1). The pPIG1b-NGGII vector (accession number AB537478), the GUS reporter plasmid for *P. patens*, was digested with *XbaI* and *BamHI.* The 3.4 kbp fragment of the *HpDTC1* promoter was inserted into the *XbaI* and *BamHI* sites of pPIG1b-NGGII using the In-Fusion Cloning Kit (Clontech, Japan) to construct the proHpDTC1::GUS vector. The proHpDTC1::GUS vector and original pPIG1b-NGGII vector were linearized by *sse83871* (Takara Bio, Japan). Transformation of *P. patens* with the polyethylene glycol method was performed as described previously^38^. Blasticidin-resistant plants were cultured for an additional week without antibiotics, and blasticidin resistance of the moss on the selection medium was confirmed. The appropriate integration of the proHpDTC1::GUS reporter and promoter-less pPIG1b-NGGII at the pPIG1b genome region was confirmed by PCR using appropriate primer sets and Southern gel-blot hybridization^40^.

### Histochemical and Quantitative GUS Measurements

For GUS histochemical analysis, *P. patens* plants were transferred to a GUS-staining buffer (100 mM sodium phosphate, pH 7.0, 10 mM EDTA, 0.5 mM K_4_Fe(CN)_6_, 0.5 mM K_3_Fe(CN)_6_, and 0.1% Triton X-100) containing 1 mM 5-bromo-4-chloro-3-indolyl-β-d-glucuronide (X-Gluc), and then incubated at 37 °C until sufficient staining was achieved. For quantitative measurement for GUS activity, *P. patens* gametophores (n = 10–15) were homogenized in an extraction buffer as previously described^41^. After centrifugation, GUS activity was fluorophotometrically measured with 1 mM 4-methyl umbelliferyl-β-D-glucuronide as a fluorogenic substrate at 37 °C.

## Additional Information

**How to cite this article**: Okada, K. *et al.* HpDTC1, a Stress-Inducible Bifunctional Diterpene Cyclase Involved in Momilactone Biosynthesis, Functions in Chemical Defence in the Moss *Hypnum plumaeforme*. *Sci. Rep.* **6**, 25316; doi: 10.1038/srep25316 (2016).

## Supplementary Material

Supplementary Information

## Figures and Tables

**Figure 1:**
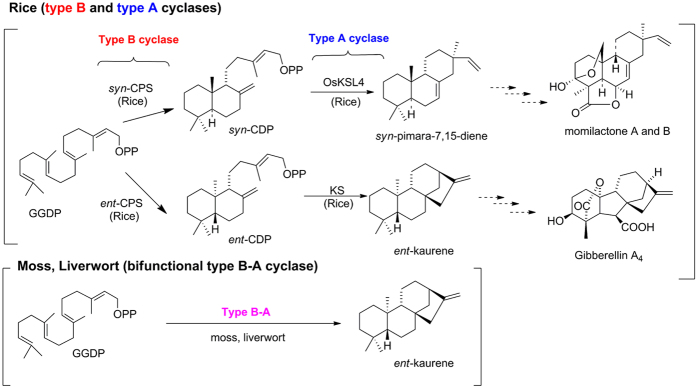
The biosynthetic pathway of diterpenoids in plants. Proposed biosynthetic pathway for momilactones in rice. Both *syn*-pimara-7,15-dine and *ent*-kaurene, the key intermediates for momilactone and gibberellin, respectively, are synthesized from geranylgeranyl diphosphate (GGDP) by sequential enzymatic reactions of type B and type A cyclases. In mosses and liverworts, cyclic diterpenes, such as *ent*-kaurene, are constructed by a bifunctional cyclase with type B and A activity in a single polypeptide.
