# Supplementary material for: HpDTC1, a Stress-Inducible Bifunctional Diterpene Cyclase Involved in Momilactone Biosynthesis, Functions in Chemical Defence in the Moss Hypnum plumaeforme
Source: Sci Rep. 2016 May 3;6:25316. doi: 10.1038/srep25316 (PMC4853780; doi:10.1038/srep25316)
Supplement: Supplementary Information [file srep25316-s1.pdf]

Supplemental Figures and table

HpDTC1, a Stress-Inducible Bifunctional Diterpene Cyclase Involved in Momilactone Biosynthesis, Functions in Chemical Defence in the Moss Hypnum plumaeforme

Kazunori Okada[1#], Hiroshi Kawaide[2#], Koji Miyamoto[3], Sho Miyazaki[2], Ryosuke Kainuma[2], Honoka Kimura[2], Kaoru Fujiwara[1], Masahiro Natsume[2], Hideaki Nojiri[1], Masatoshi Nakajima[4], Hisakazu Yamane[3], Yuki Hatano[5], Hiroshi Nozaki[5]*, and Ken-ichiro Hayashi[5]*

[#]These authors equally contributed.

*corresponding authors

Affiliations

[1] Biotechnology Research Center, The University of Tokyo, Tokyo, 113-8657, Japan.

[2] Graduate School of Agriculture, Tokyo University of Agriculture and Technology, 3-5-8 Saiwaicho, Fuchu, Tokyo 183-8509, Japan

[3] Department of Biosciences, Teikyo University, 1-1 Toyosatodai, Utsunomiya, 320-8551, Japan.

[4] Department of Applied Biological Chemistry, The University of Tokyo, 1-1-1 Yayoi, Bunkyo-ku, Tokyo 113-8657, Japan

[5] Department of Biochemistry, Okayama University of Science, 1-1 Ridai-cho, Okayama 700-0005, Japan.

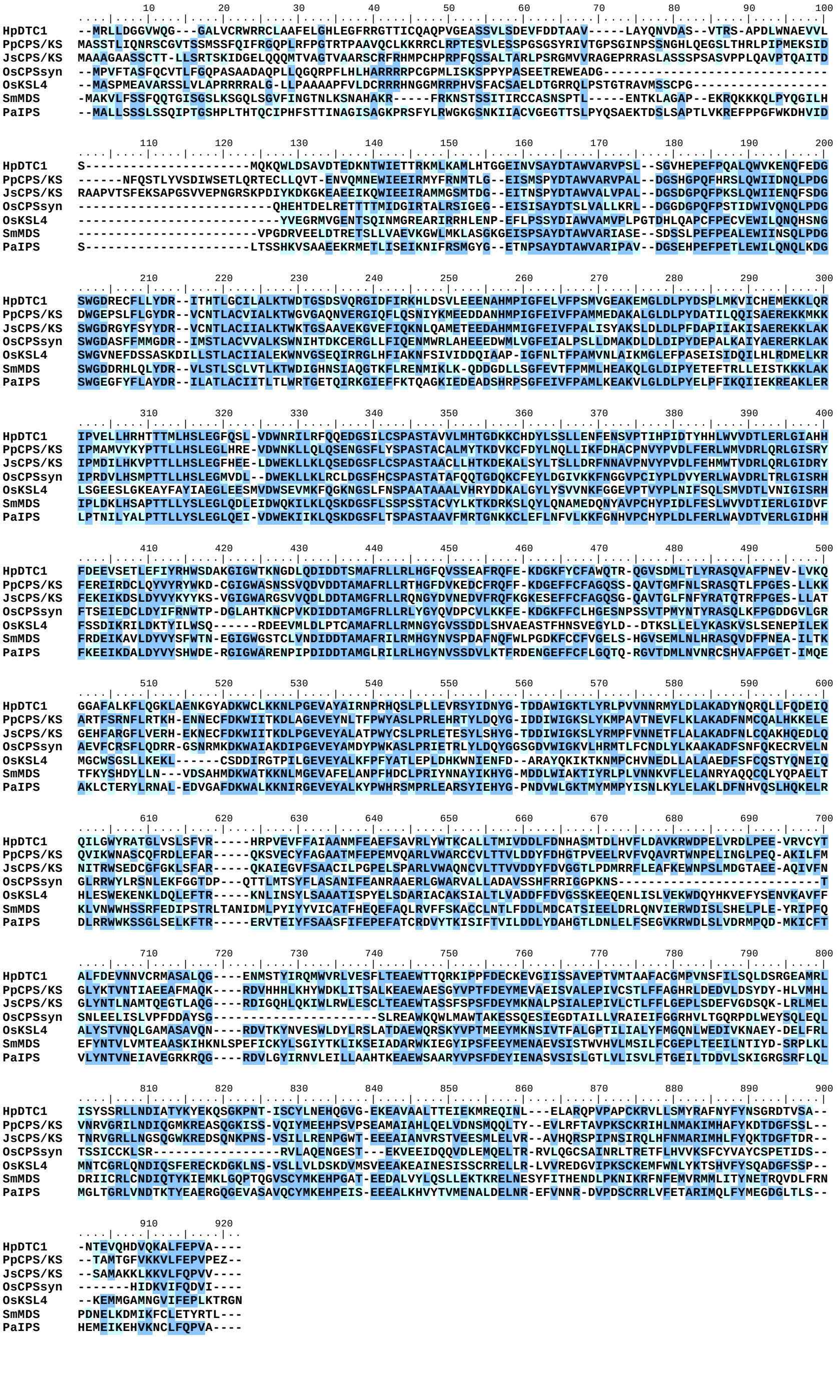

Supplementary Figure S1. Amino acid sequence alignment of HpDTC1

Moss bifunctional *ent*-kaurene synthase (PpCPS/KS: AB302933.1), liverwort bifunctional *ent*-kaurene synthase (JSCPS/KS: BAJ39816), lycophyte bifunctional miltiradiene synthase (SmMDS:BAL41682), rice monofunctional *syn*-pimaradiene synthase (OsKSL4: Q0JEZ8) and gymnosperm bifunctional isopimaradiene synthase (PaIPS: ADZ45512).

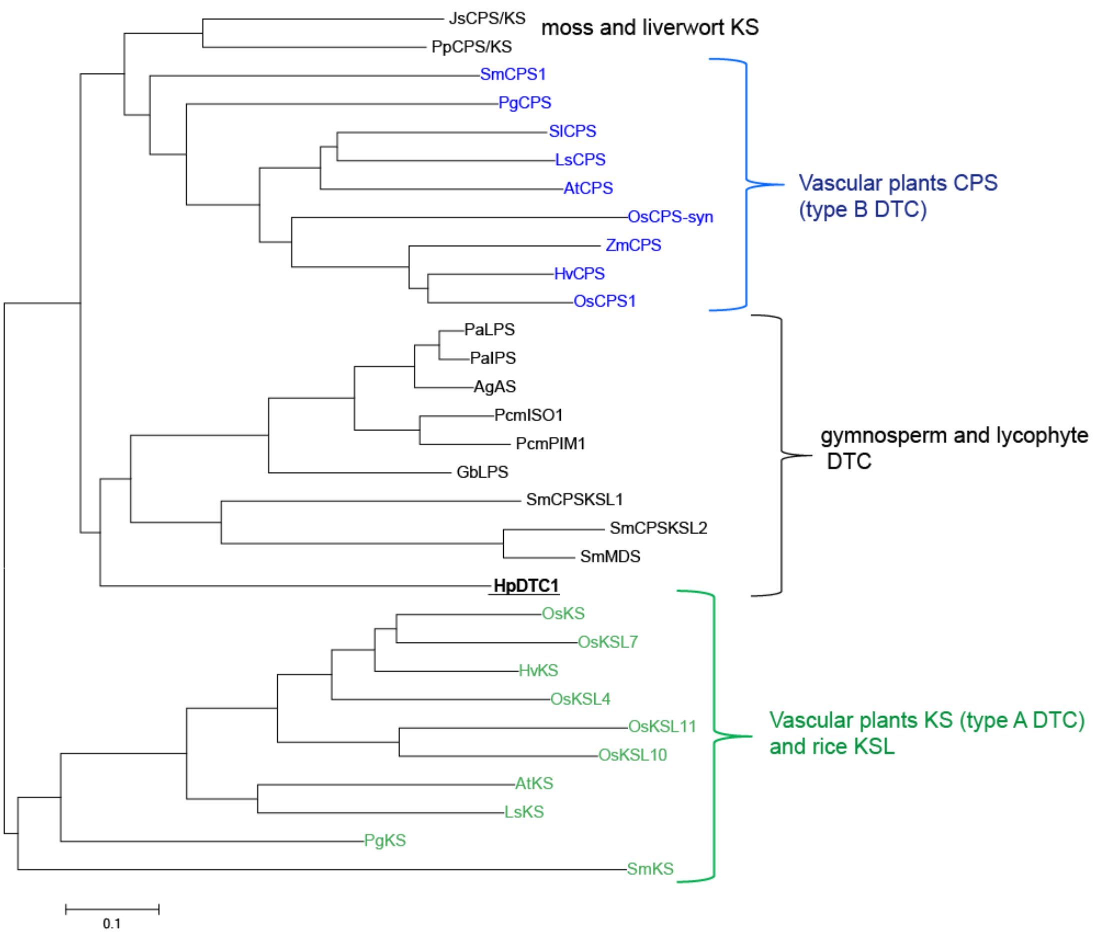

Supplementary Figure S2. Phylogenetic tree of plant diterpene cyclases.
The tree was constructed using the bootstrap neighbour joining method from alignment with the ClustalW program. HpDTC1 belongs to the gymnosperm and lycophyte bifunctional diterpene cyclases for specialized diterpene biosynthesis.

	ACCESSION	Name
PpCPS/KS	AB302933.1	ent-kaurene synthase [Physcomitrella patens]
JsCPS/KS	BAJ39816	ent-kaurene synthase [Jungermannia subulata]
SmCPS1	J9QS23	Copalyl diphosphate synthase 1, SmTPS9
PgCPS	ADB55707	ent-copalyl diphosphate synthase [Picea glauca]
SlCPS	NP_001234008	copalyl diphosphate synthase [Solanum lycopersicum]
LsCPS	BAB12440	copalyldiphosphate synthase No1 [Lactuca sativa]
AtCPS	NP_192187	Ent-copalyl diphosphate synthase [Arabidopsis thaliana]
OsCPS-syn	BAD42451	syn-CDP synthase [Oryza sativa Japonica Group]
ZmCPS	AAT70083	ent-copalyl diphosphate synthase [Zea mays]
HvCPS	AAT49065	copalyl diphosphate synthase-like protein [Hordeum vulgare]
OsCPS1	BAD42449	ent-copalyldiphosphate synthase [Oryza sativa]
PaLPS	ADZ45517	levopimaradiene/abietadiene synthase [Picea sitchensis]
PaIPS	ADZ45512	isopimaradiene synthase [Picea sitchensis]
AgAS	AAK83563	abietadiene synthase [Abies grandis]
PcmISO1	M4HYP3	Monofunctional isopimaradiene synthase [Pinus contorta]
PcmPIM1	M4HYC8	Monofunctional pimaradiene synthase [Pinus banksiana]
GbLPS	Q947C4	Levopimaradiene synthase [Ginkgo biloba]
SmCPSKSL1	AEK75338	labda-7,13E-dien-15-ol synthase [Selaginella moellendorffii]

SmCPSKSL2	BAP19109	sandaracopimaradiene synthase [Selaginella moellendorffii]
SmMDS	BAL41682	miltiradiene synthase [Selaginella moellendorffii]
OsKS	AAQ72559	ent-kaurene synthase 1A [Oryza sativa]
OsKSL7	Q00G37	Ent-cassa-12,15-diene synthase [Oryza sativa]
HvKS	AAT49066	ent-kaurene synthase-like protein 1 [Hordeum vulgare]
OsKSL4	Q0JEZ8	Syn-pimara-7,15-diene synthase [Oryza sativa]
OsKSL11	Q1AHB2	Stemod-13(17)-ene synthase [Oryza sativa]
OsKSL10	ABH10735	ent-sandaracopipmaradiene synthase [Oryza sativa]
AtKS	AAC39443	ent-kaurene synthase [Arabidopsis thaliana]
LsKS	BAB12441	ent-kaurene synthase No1 [Lactuca sativa]
PgKS	ADB55711	ent-kaurene synthase [Picea glauca]
SmKS	BAP19110	ent-kaurene synthase [Selaginella moellendorffii]

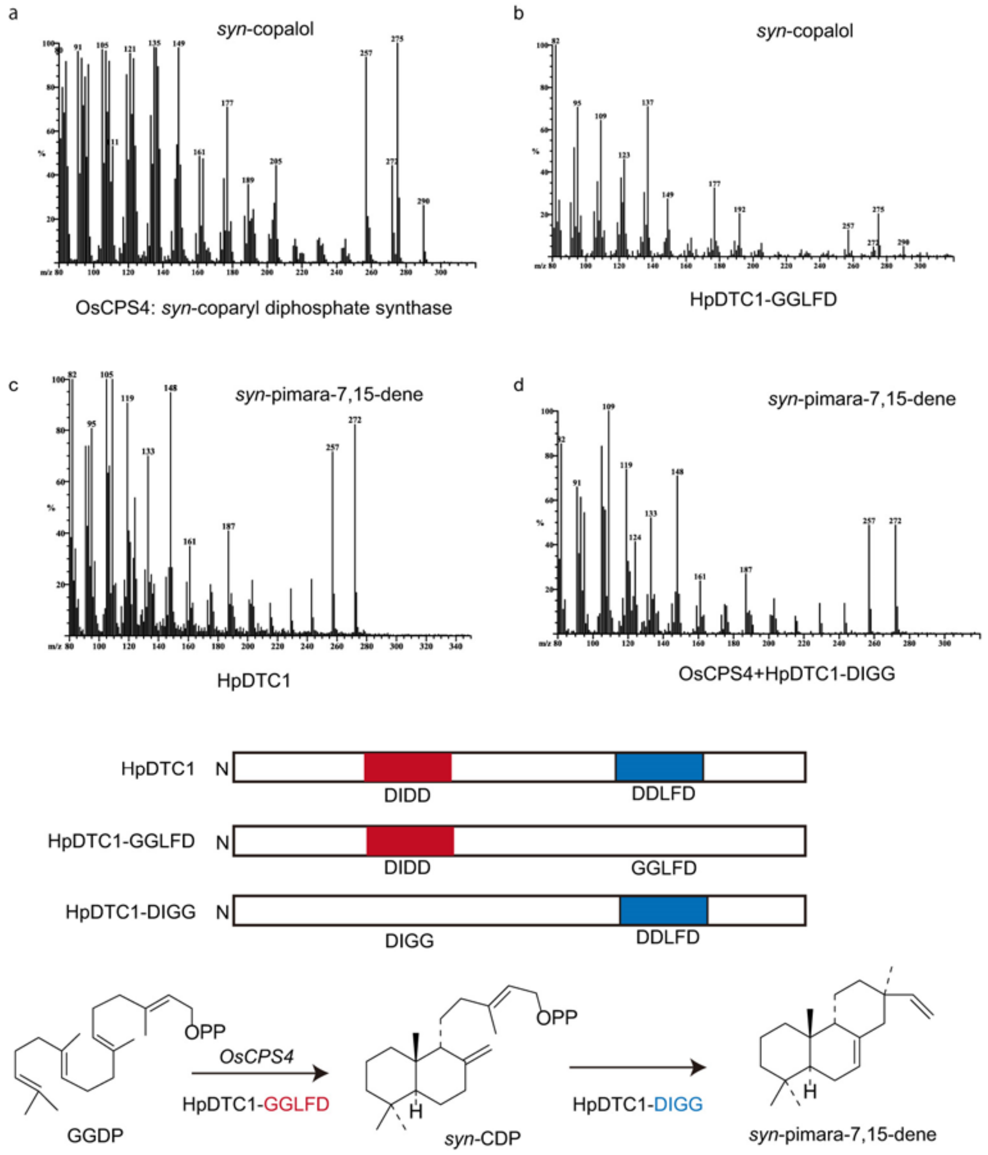

Supplementary Figure S3. GC-MS spectra of enzymatic products of recombinant HpDTC1 mutant enzymes.

(a) *syn*-copalol, product of OsCPS4. (b) The product of the HpDTC1-GGLFD mutant enzyme was identical to *syn*-copalol. (c) *syn*-pimara-7,15-diene, HpDTC1 product. (d) The reaction product from OsCPS4 and the HpDTC1-DIGG mutant enzyme was identical to *syn*-pimara-7,15-diene. The reaction product, *syn*-CDP, in (a and b) was dephosphorylated by calf intestinal alkaline phosphatase.

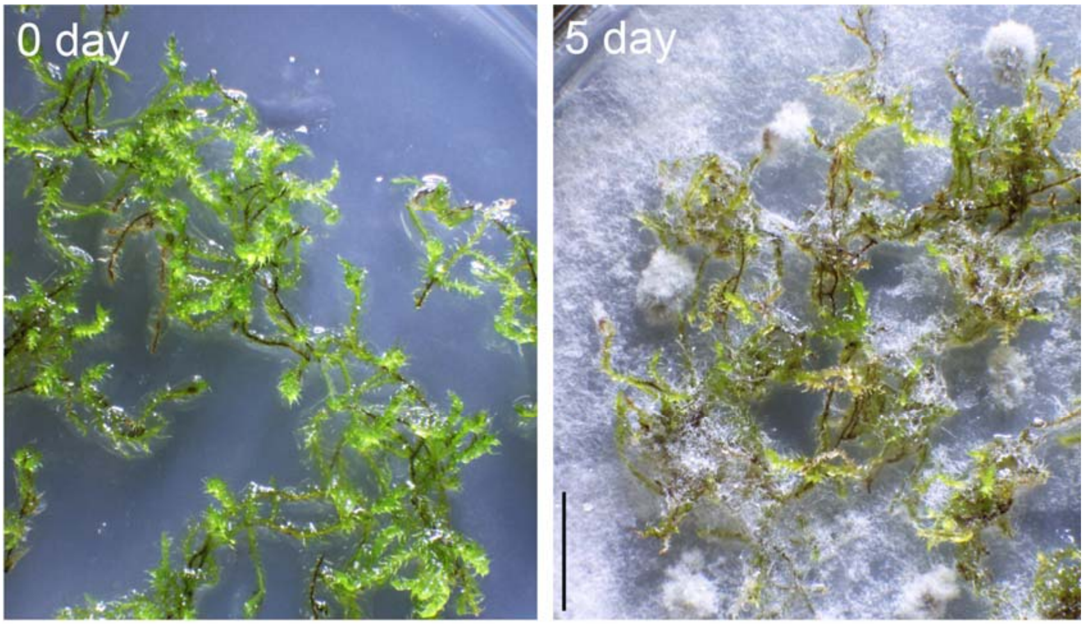

Supplementary Figure S4. Photograph of *Botrytis cinerea* infected *Hypnum plumaeforme*
Hypnum plumaeforme was cultured at 24 ºC for 5 days after inoculation with *Botrytis cinerea* mycelia. Scale bar: 10 mm.

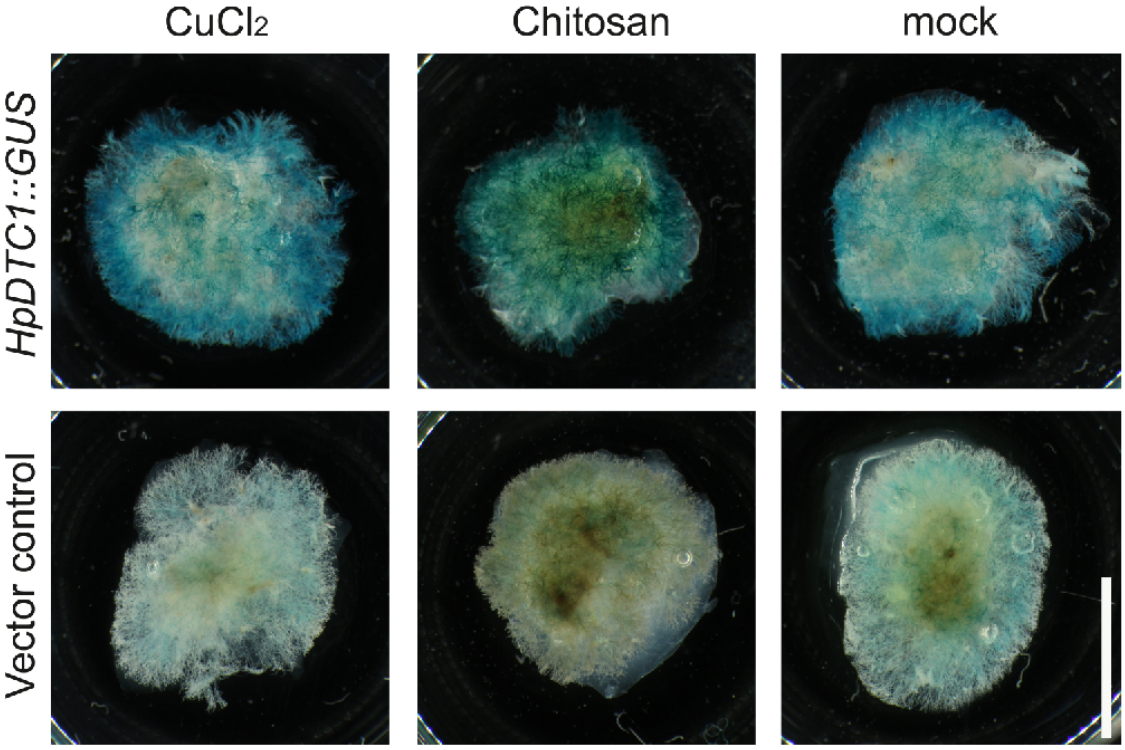

Supplementary Figure S5. ***HpDTC1::GUS*** **reporter gene expression in protonema cells of *P. patens*.** Protonema colonies of *HpDTC1::GUS* and vector control lines were incubated with 500 µM $CuCl_2$ and chitosan (500 µg/ml) for 24 h and then histochemically stained with X-Gluc. Scale bar: 5 mm.

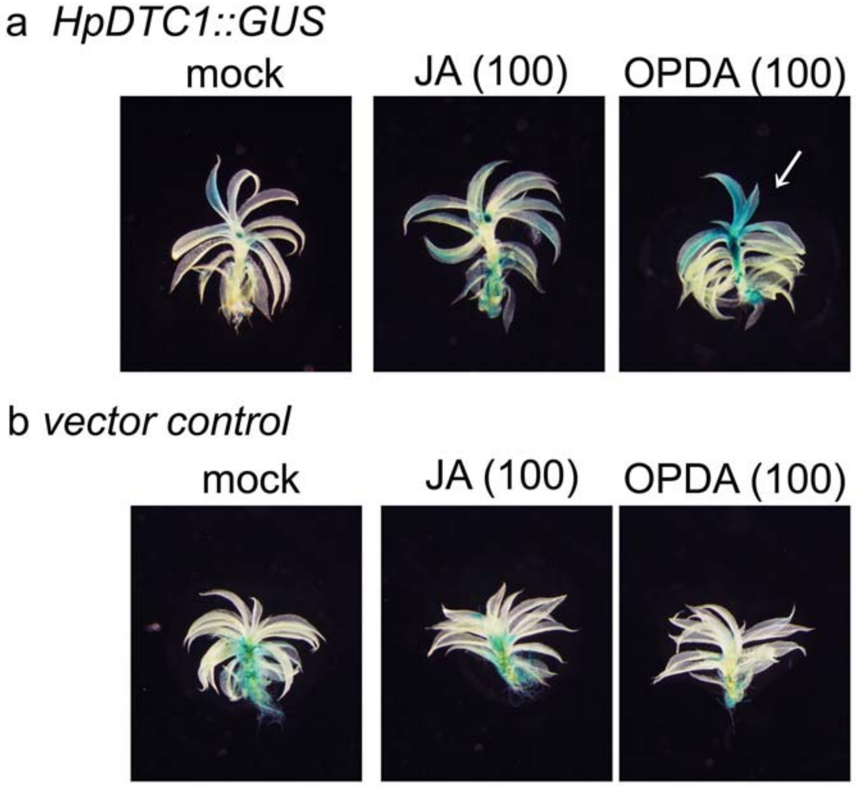

Supplementary Figure S6. The effects of jasmonate and 12-oxo-phytodienoic acid on *HpDTC1::GUS* expression.

After 24 h treatment with jasmonate (JA) and 12-oxo-phytodienoic acid (OPDA), *HpDTC1::GUS* reporter expression was visualized by X-gluc. (a) OPDA induced *HpDTC1::GUS* reporter expression, but JA did not. (b) JA and OPDA did not affect GUS expression in the control line. The values in parentheses represent the concentration (μM).

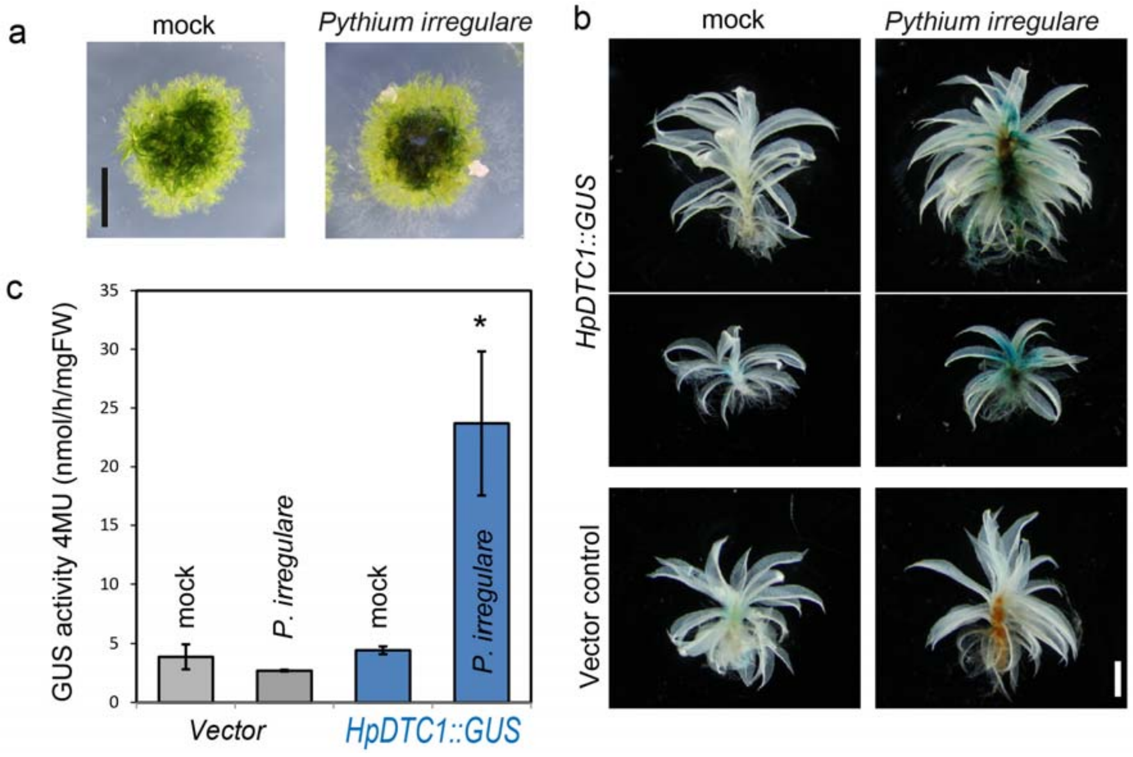

Supplementary Figure S7. The *HpDTC1::GUS* reporter gene was induced by *Pythium irregulare* infection in *P. patens* gametophores.

(a) Mycelia of *Pythium irregulare* MAFF 237501 were inoculated onto colonies of *HpDTC1::GUS* and vector control lines and then plates were incubated for 4 days. Scale bar: 5 mm. (b) The *HpDTC1::GUS* plants were histochemically stained by X-gluc. Scale bar: 1 mm. (c) The quantitative data for *HpDTC1::GUS* reporter induction by *P. irregulare* infection after 4 days incubation. The GUS activity was fluorometrically determined. Data are presented as mean ± standard deviation of biological replicates. (*n*=5-8, *$p < 0.01$.).

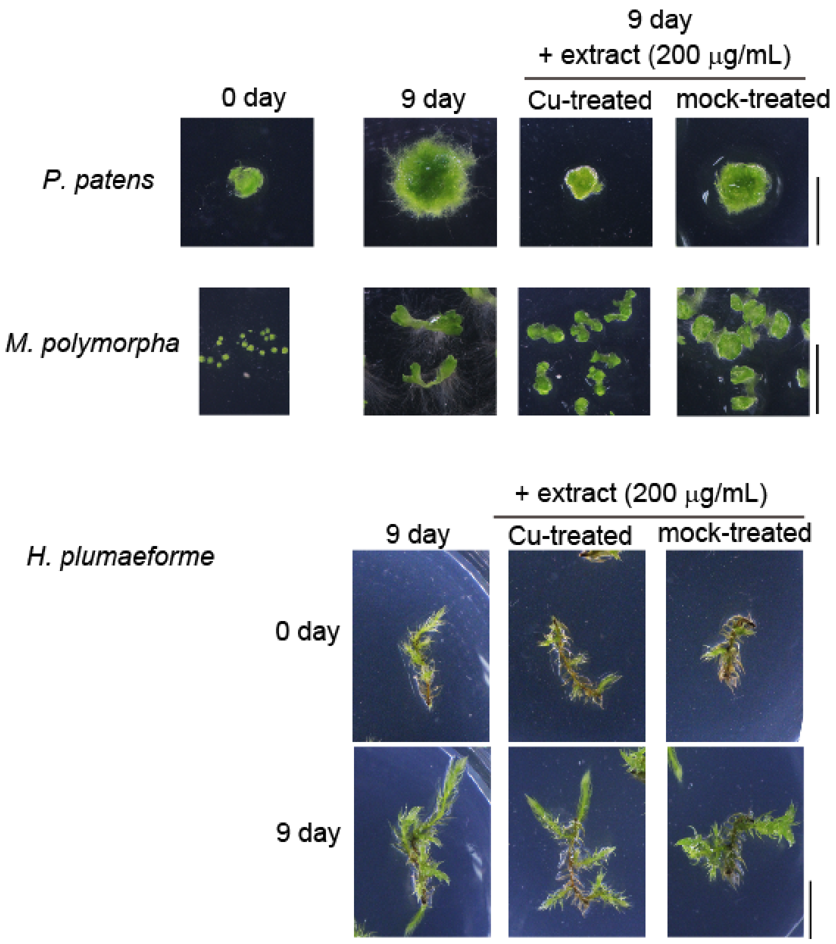

Supplementary Figure S8. Allelopathic activity of *H. plumaeforme* extracts on the growth of the moss *P. patens* and liverwort *M. polymorpha.*

The acetone extract was prepared from *H. plumaeforme* gametophores treated with or without 500 μM $CuCl_2$. The plants were cultured on BCDATG agar medium containing the extract (200 μg/mL) and then photographs were taken on the indicated days. Scale bars: 5 mm.

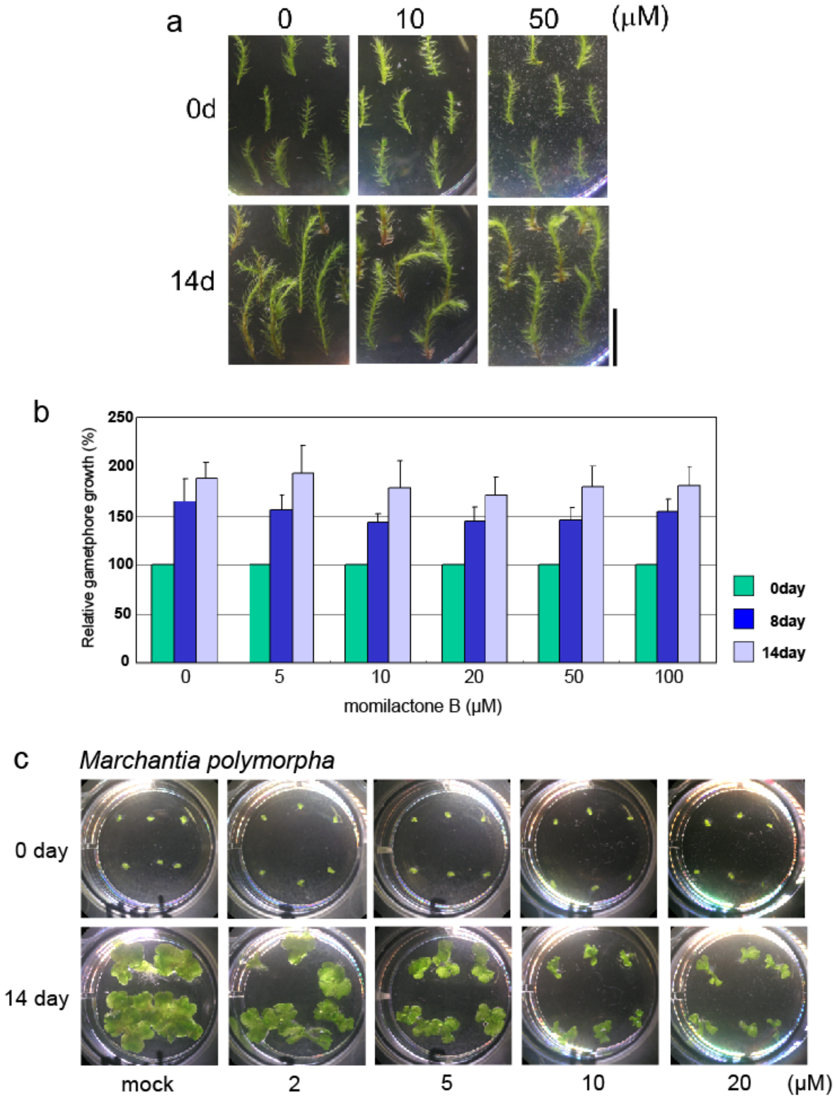

Supplementary Figure S9. Allelopathic activity of momilactone B on the growth of *H. plumaeforme* and liverwort *M. polymorpha*.

(a) Photograph of *H. plumaeforme* gametophores grown on BADATG agar plates containing momilactone B (10 and 50 μM). (b) The growth of H. *plumaeforme* was recorded after 8 and 14 days incubation. The values shown are the relative length of gametophores at 0 days (100%). Inhibitory effects of momilactone B on the growth was not observed at 100 μM. (c) *M. polymorpha* was grown on M51 agar medium containing momilactone B and cultured for 14 days. Momilactone B showed potent inhibitory activity on the growth of *M. polymorpha* at 2-5 μM.

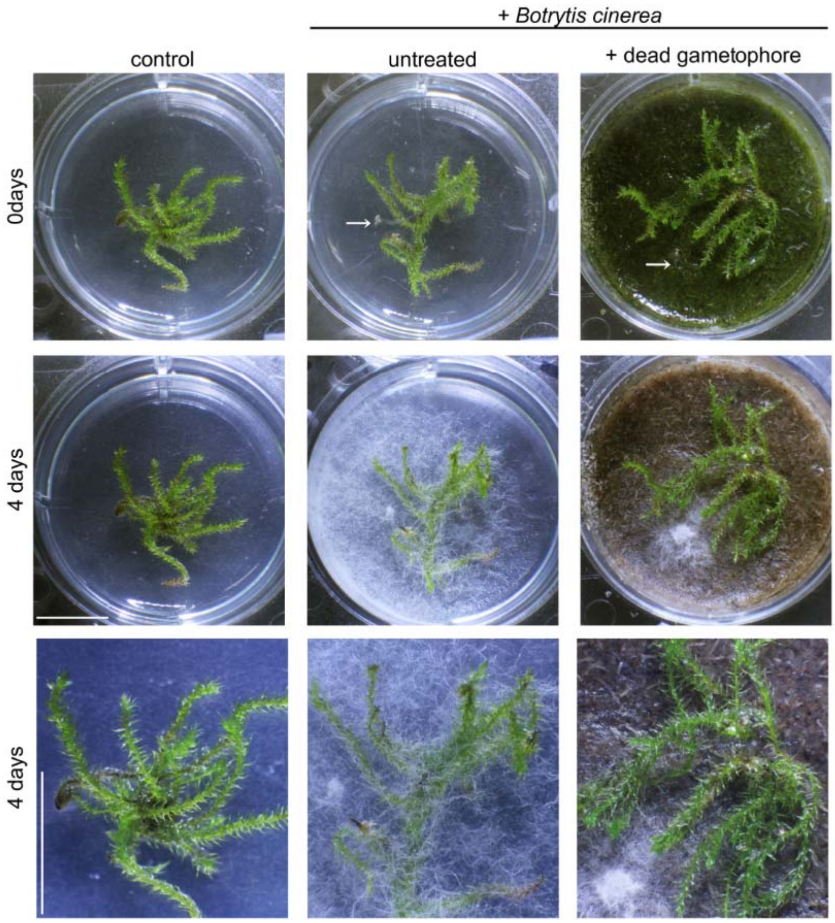

Supplementary Figure S10. The effects of dead gametophytes of *H. plumaeforme* on the growth of *B. cinerea.*

Mycelia were inoculated onto BCDATG agar plates containing the dried gametophores of *H. plumaeforme* (10% w/v) and then fresh *H. plumaeforme* gametophores were placed on the plate. The photograph was taken after 4 days incubation. Scale bar: 10 mm.

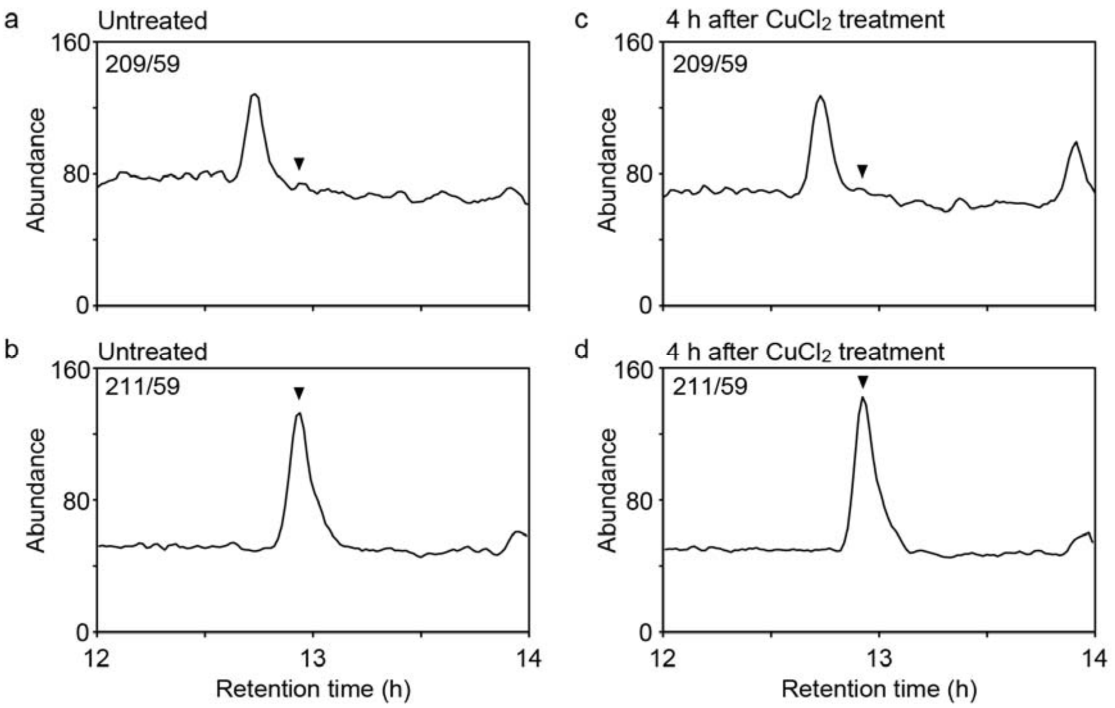

Supplementary Figure S11. Measurements of endogenous jasmonic acid in *H. plumaeforme.*

Moss samples (about 100 mg fresh weight) were homogenized and suspended in 2 mL 80% (v/v) methanol. After adding 5 ng [$^2H_2$]-JA as an internal standard, the supernatant was loaded onto a Bond Elut C18 column (100 mg, 3 mL; Agilent, CA), which had been sequentially pre-washed with 3 mL each of methanol and water and equilibrated with 3 mL 80% (v/v) methanol, which was followed by elution with 2 mL 80% (v/v) methanol. The flow-through and eluted fractions were collected together and concentrated to a volume of about 0.5 mL *in vacuo*. The concentrated samples were subjected to liquid chromatography with electrospray ionization tandem mass spectrometry (LC-ESI-MS/MS) to quantify JA accumulation as previously described in Tamiru et al., A chloroplast-localized protein, LESION AND LAMINA BENDING, affects defence and growth responses in rice. *New Phytol*. 2016 *doi: 10.1111/nph.13864*. (a, c) SEM chromatogram (*m/z* 209>59) corresponding to the fragment ion from endogenous JA in untreated sample (b, d) SEM chromatogram (*m/z* 211>59) corresponding to the fragment ion from the internal standard [$^2H_2$]-JA in 4h-treated sample for 4 h. A black arrow indicates the peak derived from JA. The endogenous JA level in *H. plumaeforme* was lower than the detection limit of our analysis system (<0.5 ng/gFW).

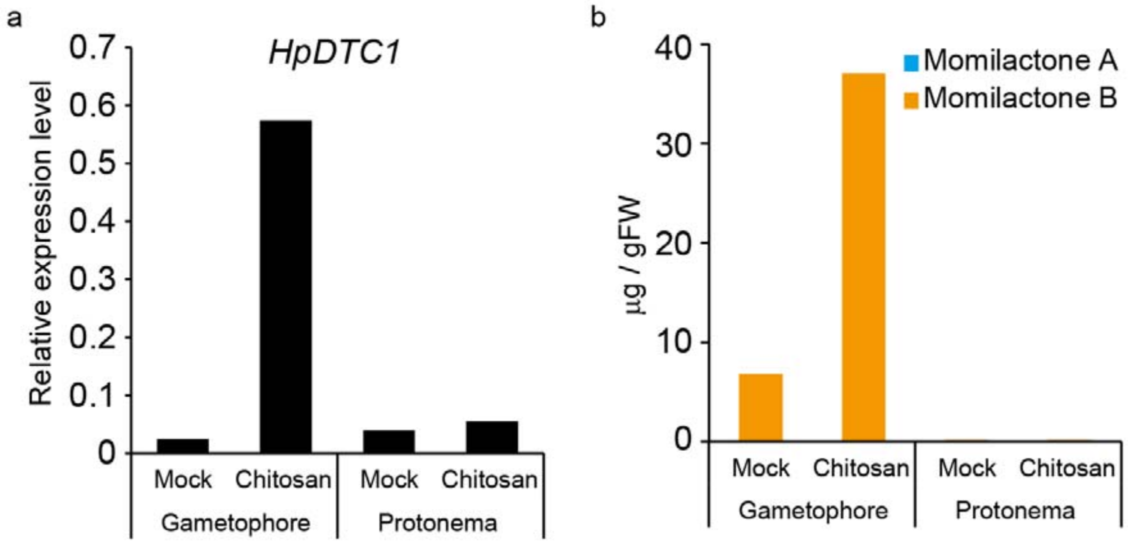

Supplementary Figure S12. *HpDTC1* expression and production of momilactones are specifically induced in the gametophores, but not in the protonema cells of *H. plumaeforme.*

(a) qRT-PCR was performed using *HpDTC1* specific primers with total RNA from *H. plumaeforme* gametophores and protonema cells treated with chitosan (500 µg/mL) for 6 h. (b) Momilactones A and B in *H. plumaeforme* gametophores and protonema cells treated with chitosan (500 µg/mL) for 6 h were measured by LC-MS/MS. In protonema cells, *HpDTC1* was not induced by chitosan treatment and consequently momilactones did not accumulate.

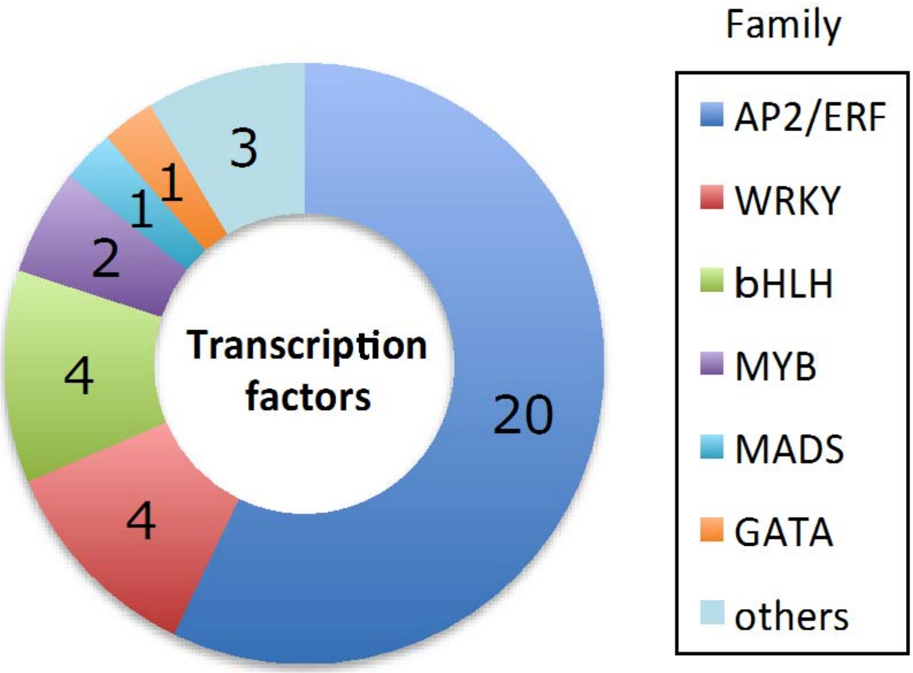

Supplementary Figure S13. ***H. plumaeforme*** **transcription factors induced by $CuCl_2$ treatment.** Forty-two genes up-regulated more than 5-fold compared to untreated samples are categorized by types of families.

Supplementary table S1 List of PCR primers

HpDTC1_cDNA internal fragment	HpDTC1-FR1-Fd	AGAAGACAAGAATACCTGGATAGAGACGAC
	HpDTC1-FR1-Rv	TTTGGACATCGTGCTGGACTTCA
HpDTC1-RACE GSP primer for 5'-end	HpDTC2-RACE5'-116	GGAAACTCGGGTTCGTGAACTCCAGATAGC
	HpDTC2-RACE5'-266	TCTTTGCACACTGTCACTTCCCGTGTCCC
HpDTC1-RACE GSP primer for 3'-end	HpDTC2-RACE3'-1929	TGTCGCAACTGGATTCCCGTGGTGAAGC
	HpDTC2-RACE3'-2098	CGGCATTGACTACTGAAATCGAGAAGATGC
Cloning for HpDTC1_ORF	HpDTC1_cDNA Fd	TTATCGATGCAAAAGCAGTGGTTAG
	HpDTC1_cDNA Rv	AACGAACATTTTTGGGACTCTGGAACGCA
TAIL AD primers	TAIL-P1	NGTCGASWGANAWGAA
	GSP1-1	TAGAGTGTGAGTGATTCTGTCGTAA
TAIL GSP primers	GSP1-2	CCCAAGAACCATCTTCAAACTG
	GSP1-3	CATTGCAGAGCTTGAGGAAACT
TAIL AD primers	TAIL-P2	GTNCGASWCANAWGTT
	GSP2-1	TGAACCTACGAAAGTATCAACCGATC
TAIL GSP primers	GSP2-2	TACATTCCGACCCAATGTGGAG
	GSP2-3	CTGCGTAAGTTTCCTCAAGCTC
TAIL AD primers	TAIL-P1	NGTCGASWGANAWGAA
	GSP3-1	ATGAGAGCTGAGTGGCCATTG
TAIL GSP primers	GSP3-2	CACGGTTGGTTCATTCCTACTTCT
	GSP3-3	TTCCATAAAGGTCAGTCGGG
TAIL AD primers	TAIL-P2	GTNCGASWCANAWGTT
	GSP4-1	CCAAGGTCATTAAGGCAAGC
TAIL GSP primers	GSP4-2	GCTCCATTGACCGATAAACA
	GSP4-3	GTGGATGATTCTATTGGTTGAGA
TAIL AD primers	TAIL-P3	WGTGNAGWANCANAGA
	GSP5-1	GAGAATTACAACAAGAGAATTACCT
TAIL GSP primers	GSP5-2	CCAAGGTCATTAAGGCAAGC
	GSP5-3	GCTCCATTGACCGATAAACA
HpDTC1 promotor cloning	PR-InF-HpDTC1-Fd	CTATAGGGGAAAGCTTAAAATAATGAAGAGTGGAGAGAGAGCTACAGT
	PR-InF-HpDTC1-Rv	GTGAATTCGAGCTCGGTACCGTCGTCTCTATCCAGGTATTCTTGTCTTC
HpDTC1-expression vector	HpDTC1-ORF-Fd	GGCATATGGAGCTCGGTACCGCCGTCGACACAGAAGACAA
	HpDTC1-ORF-Rv	CGACAAGCTTGAATTCTCAGGCCACAGGCTCGA
	double-mutation-A Fw	GAAGAGGCCGCCCACGAT
HpDTC1 mutant enzymes	double-mutation-A Rv	ATCGTGGGCGGCCCTCTTC
	double-mutation-B Fw	GGCGGTACTTCAATGGCTTTCC
	double-mutation-B Rv	TATATCTTGCAAATCACCATTCTTAGTCCATC
	HpACT3-Fd	CGAGCAGCATGAAGATCAAG
	HpACT3-Rv	GTACTCGCTCTTCGCAATCC
qRT-PCR primers	DTC1_for_RTPCR-Fd	TGCTGCTCAGCATGTATCGT
	DTC1_for_RTPCR-Rv	GGACTCTGGAACGCAAGACT
HpDTC1::GUS	PR-DTC1-GUS-Fd	ATTCATGTTTTCTAGAAAAATAATGAAGAGTGGAGAGAG
	PR-DTC1-GUS-Rv	CCGGGGGGGGGGATCCTCCCTGGACATGAAGTTCGGTGTGC
